# Reflections of the social environment in chimpanzee memory: applying rational analysis beyond humans

**DOI:** 10.1098/rsos.160293

**Published:** 2016-08-03

**Authors:** Jeffrey R. Stevens, Julian N. Marewski, Lael J. Schooler, Ian C. Gilby

**Affiliations:** 1 Department of Psychology and Center for Brain, Biology and Behavior, University of Nebraska-Lincoln, Lincoln, USA; 2 Center for Adaptive Behavior and Cognition, Max Planck Institute for Human Development, Berlin, Germany; 3 Faculty of Business and Economics, University of Lausanne, Lausanne, Switzerland; 4 Department of Psychology, Syracuse University, New York, NY, USA; 5 School of Human Evolution and Social Change, and Institute of Human Origins, Arizona State University, Tempe, AZ, USA

**Keywords:** chimpanzees, comparative cognition, forgetting, memory, rational analysis, social contact

## Abstract

In cognitive science, the rational analysis framework allows modelling of how physical and social environments impose information-processing demands onto cognitive systems. In humans, for example, past social contact among individuals predicts their future contact with linear and power functions. These features of the human environment constrain the optimal way to remember information and probably shape how memory records are retained and retrieved. We offer a primer on how biologists can apply rational analysis to study animal behaviour. Using chimpanzees (*Pan troglodytes*) as a case study, we modelled 19 years of observational data on their social contact patterns. Much like humans, the frequency of past encounters in chimpanzees linearly predicted future encounters, and the recency of past encounters predicted future encounters with a power function. Consistent with the rational analyses carried out for human memory, these findings suggest that chimpanzee memory performance should reflect those environmental regularities. In re-analysing existing chimpanzee memory data, we found that chimpanzee memory patterns mirrored their social contact patterns. Our findings hint that human and chimpanzee memory systems may have evolved to solve similar information-processing problems. Overall, rational analysis offers novel theoretical and methodological avenues for the comparative study of cognition.

## 1. Introduction

Roboticists face difficult problems when developing artificial memory systems. For one, those systems inevitably come with limited capacity—there are physical bounds to how much information can be stored. As a result, the system must have mechanisms that determine *what* information it should store and, as new information poses additional demands for storage capacities, for *how long* it should maintain older information. That is, the system must essentially make a bet about what information it will need in current and future contexts. Furthermore, the system must quickly retrieve only that information relevant to the context.

**Figure 1.**
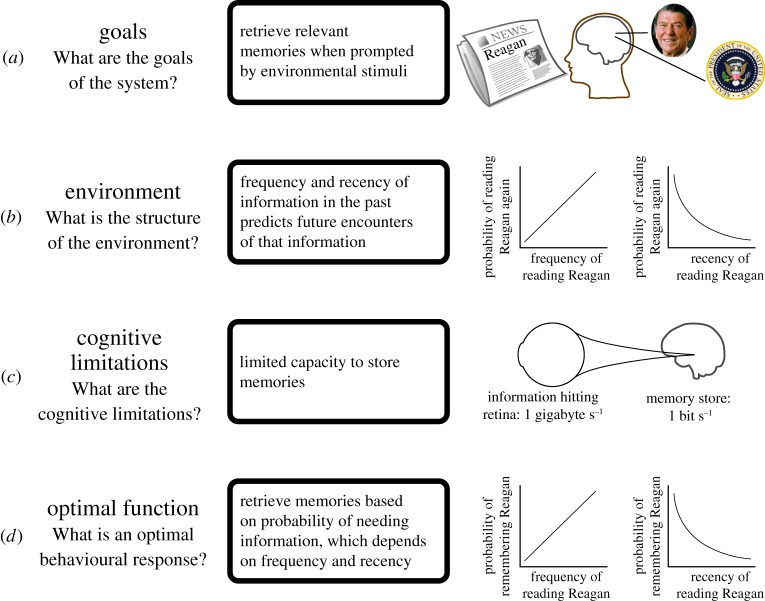
Rational analysis. Anderson [1] describes a number of key steps used by the rational analysis framework. Here, we illustrate these steps using adaptive memory as an example. (*a*) First, one must specify the goals of the cognitive system, which, for memory, involves retrieving information based on triggers in the environment. For example, reading the word ‘Reagan’ should trigger memories of the person Ronald Reagan, who was the President of the United States of America. (*b*) Next, one must measure the structure of the environment. How frequently and recently one encounters an object in the environment (e.g. reading the word ‘Reagan’) strongly predicts the probability of encountering that object again, which is relevant for memory storage and retrieval. (*c*) Cognitive limitations of the agents must also be considered to constrain the cognitive system. Given the sheer amount of information flowing into cognitive systems, storage capacity is a severe limitation of memory. Humans are estimated to have an 11 billion-fold loss of information from the retina to memory storage (information flow rates from [2]). (*d*) Finally, one must derive the optimal behavioural response to the environmental structure based on the constraints on the system. For memory, frequency and recency of encounter with objects in the environment should predict the probability of needing information about that object in the future. These steps can be iterated to refine the theory.

Biological information-processing systems face the same problems. Most notably, in coping with biological (e.g. storage, computational) constraints, human memory must encode, maintain, prioritize and recall information that is relevant to current or future stimuli in the environment, while removing irrelevant records. How can such systems be best designed? In humans, one possibility is that the cognitive system could take advantage of statistical regularities in the environment—a thesis that, in cognitive science, has been prominently advanced by Anderson's [1] *rational analysis*. This framework proposes that cognition is an adaptive response to recurrent environmental structures and provides a step-by-step approach to investigate how cognition solves those information-processing problems. Specifically, rational analysis specifies (figure 1):

— what goals cognitive systems need to achieve;
— how the environment in which the systems act is structured;
— what computational constraints the systems face; and
— what cognitive processes would best achieve the goals given environmental structure and cognitive limitations.

For instance, by mimicking general statistical regularities in human social, linguistic and other environments, human forgetting and recall processes pose optimal solutions to the adaptive problems of storing relevant information, retrieving that information when needed and forgetting those aspects of it that are most likely irrelevant or outdated [3,4].

Anderson and co-workers' [1,3–5] initial work on memory has since inspired research into other important aspects of cognition such as whether humans optimally categorize objects in the environment [6–8], optimally allocate cognitive and motor resources when interacting with objects in the world [9] or represent numerical information [10]. Indeed, rational analysis has become a well-established methodological framework in cognitive science [11], though some have criticized optimization models of decision making because actual optimization in many real-world environments is infeasible [12].

Though biologists are often interested in how the environment shapes behaviour, they tend to focus on the first components of rational analysis: adaptive goals and the structure of the environment [13–16]. Cognitive processes and computational limits that shape how those adaptive goals are achieved, tend to be under-studied in biology. Using our closest living phylogenetic relative—the chimpanzee (*Pan troglodytes*)—as a case study, we offer a methodological primer of how biologists can use the rational analysis approach to study how statistical patterns in the environment can shape cognitive and behavioural systems across species. Specifically, we start by reviewing how cognitive scientists have applied rational analysis to understanding human memory and environments. We then apply the rational analysis framework by analysing chimpanzee environments and memory. We end by discussing implications of our findings for the evolution of cooperation, cultural transmission, disease transmission and cognitive evolution more generally.

### 1.1. Human memory

Do you remember what you had for lunch yesterday? How about last week or last month? Human memory performance declines rapidly as the time between encoding and retrieving information increases [17]. Rational analysis proposes that forgetting is an adaptive response to how information is distributed in the world [3–5]. The statistical distribution of information in the environment shapes what information the cognitive system might need to achieve its processing goals. Human memory is like an Internet search engine that provides the cognitive system with important information when needed [1]. According to rational analysis, the memory system meets the informational demands arising from environmental stimuli by retrieving memories associated with these stimuli. The cognitive system tries to make available the most useful memories by acting on the expectation that environmental stimuli tend to reappear in predictable ways.

According to Anderson & Schooler [4], the cognitive system wagers that, as the recency and frequency with which an individual encounters a piece of information increase, so does the probability that this information will be needed to achieve future processing goals (henceforth: *need probability*). Conversely, the more time that has passed since the last encounter, the more likely that memories associated with the stimulus will not be needed and therefore can be forgotten. Memory, therefore, sets aside outdated, irrelevant information, prioritizing retrieval of frequently and recently encountered—and thus more relevant—information. As processing unneeded information comes with (e.g. metabolic) costs, the cognitive system performs more efficiently by eliminating such information. Therefore, the accuracy of retrieving memories about an object in the environment should depend on the *frequency* and *recency* of encounters with that object.

Anderson and co-workers [18] used the rational analysis of memory to guide the development of a memory system that is part of the ACT-R (Adaptive Control of Thought--Rational) theory of cognition. ACT-R's memory system tags pieces of information with activation values that govern the accessibility of information, that is, whether and how quickly it can be retrieved. In other words, these activations reflect the need probability, *p,* from the rational analysis of memory. More formally, the log odds (i.e. ln[*p*/(1 – *p*)]) that a piece of information will be needed to achieve a processing goal is rooted in its environmental pattern of occurrence. Specifically, activation, *A*, is determined by an equation that strengthens and decays a piece of information's activation according to the pattern with which it has been needed

1.1 $$A=\ln\sum_{k=1}^{n}t_{k}^{-d}=\ln\left(\frac{p}{1-p}\right),$$

where the information has been needed *n* times in the past at time lags of *t*_1_, *t*_2_, … , *t*_*n*_. Finally, *d* is a decay parameter that captures the amount of forgetting, thus determining how much information about an object's environmental frequency is retained in memory. Across a wide variety of tasks and contexts, this equation does a remarkable job of predicting memory accuracy and corresponding neural markers in the brain [18–20].

This activation equation captures the relationships between memory accuracy and frequency and recency of encounter that have been carefully measured by experimental psychologists. Memory researchers measure frequency by counting the number of encounters with an object and recency by tracking the amount of time since the object was last encountered. In his classic 1913 study, Ebbinghaus [17] provided early data on how frequency and recency influenced his own recall of nonsense syllables. He found that his memory accuracy increased with the frequency with which he practised the syllables. Specifically, memory accuracy increased with frequency as a linear function: *P* *=* *an* *+* *b,* where *P* represents the probability of future encounter, *n* represents the number (frequency) of prior encounters, and *a* and *b* represent free parameters.

Recency also predicted memory accuracy, but, rather than a linear relationship, it followed a negatively accelerating function. Since then, memory researchers have tested two competing functions that may account for this relationship: a power function, *P* *=* *ct^−*d*^*, where *t* represents the amount of time that has passed since the last encounter (recency), and *c* and *d* represent free parameters, and an exponential function, *P* *=* *ce^−^*^*dt*^. There is controversy in the literature regarding whether exponential or power-law functions best capture forgetting. Nevertheless, many researchers favour power-law functions for the relationship between memory and recency on both theoretical and empirical grounds [4,21–23].

Thus, human memory performance demonstrates key regularities based on how objects are encountered in the environment. The frequency and recency of encounters with information predict memory accuracy with linear and power-law functions, respectively.

### 1.2. Human environment

Anderson & Schooler [4] hypothesized that memory performance reflects the statistical regularities with which stimuli are encountered in the environment. With the memory data in hand, they explored this hypothesis by measuring frequency and recency of encountering words in two linguistic environments: children's verbal interactions and headlines in *The New York Times*. In *The New York Times* example, reading a word (e.g. Reagan) in a headline warrants retrieving information about the associated concept (e.g. the President of the United States of America). Likewise, whenever someone speaks to a child, the child must retrieve the word's meaning.

**Figure 2.**
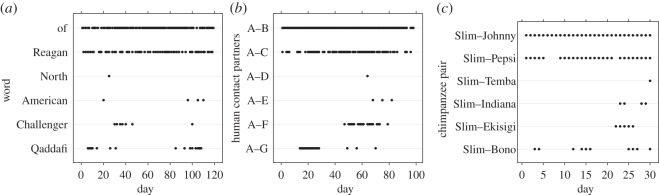
(*a*) Pattern of word usage in *The New York Times* over a 120 day period. Adapted with permission from Anderson & Schooler [4]. (*b*) Pattern of encounters between pairs of human participants over a 100 day period. Graph based on data from Pachur *et al*. [24]. (*c*) Pattern of encounters between pairs of wild chimpanzees over a 30 day period.

**Figure 3.**
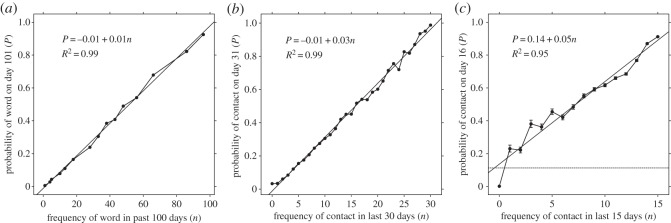
In humans and chimpanzees, the frequency of past interactions linearly predicts the probability of future interactions. (*a*) The frequency of a word appearing in *The New York Times* headlines in the previous 100 days predicts the probability of the word appearing again on day 101. Adapted with permission from Anderson & Schooler [4]. (*b*) The frequency of a human participant encountering a social partner in the previous 30 days predicts the future probability of contact with that partner. Adapted with permission from Pachur *et al*. [24]. (*c*) The frequency of a chimpanzee encountering a social partner in the previous 15 days predicts future contact with that partner. Errors bars show binomial 95% CIs. Dashed line represents overall mean probability of contact in the dataset.

**Figure 4.**
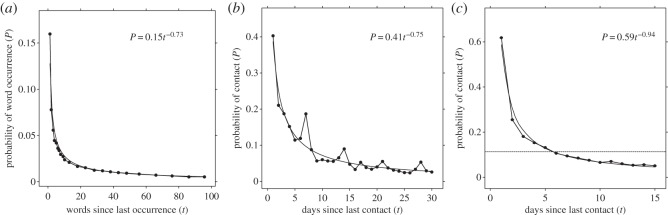
In humans and chimpanzees, the recency of past encounters predicts the probability of future encounters consistent with a power-law function. (*a*) The recency of a word appearing in *The New York Times* headlines predicts the probability of the word appearing again. Recency is measured as the number of other words since the last occurrence of the target word. Adapted with permission from Anderson & Schooler [4]. (*b*) The recency of a human participant encountering a social partner predicts the future probability of contact with that partner. Adapted with permission from Pachur *et al*. [24]. (*c*) The recency of a chimpanzee encountering a social partner predicts future contact with that partner. Error bars with binomial 95% CIs are too small to plot. Dashed line represents overall mean probability of contact in dataset. Curves illustrate best fitting power functions.

In these two linguistic environments, the probability of encountering a particular word depended on the frequency and recency of encounter [4]. To illustrate the point, figure 2*a* shows the pattern of word usage over a 120 day period in *The New York Times*. As they did in Ebbinghaus's [17] memory data, the probability of encountering a word increased linearly with the frequency of prior encounter (figure 3*a*) and decreased as a power function with the recency of encounters (figure 4*a*). That is, the statistical relationships of both written and oral word usage in the environment matched the statistical relationships of memory for words.

Anderson & Schooler's [4] findings generalize beyond word usage to social contact data, as well. Encountering a member of one's social network often involves retrieval of information about relationship status with that individual (e.g. relatedness, dominance status) or his/her past behaviour. For instance, before attempting to engage a social partner in a cooperative interaction, it is probably useful to remember whether the partner cooperated previously [25]. In an uncertain and variable world, larger and more recent samples of information are more accurate [26] and information becomes out of date over time [27,28]. Encountering social partners frequently and recently can provide more accurate predictions about future behaviour than less frequent and older information; therefore, frequency and recency of social contact are important but under-studied components of the social environment. Anderson & Schooler and Pachur *et al*. [24] explicitly tested how frequency and recency of past social contact predicted future contact. Anderson & Schooler used Anderson's personal e-mail usage, and Pachur *et al*. recorded face-to-face, phone, e-mail and other personal interactions in a sample of 40 people over 100 days. Both datasets exhibited the same lawful linear and power relationships observed in the linguistic environments. Figure 2*b* shows examples of spacing patterns that match those of word usage in *The New York Times*. Further, the frequency and recency of social encounters predicted future encounters in linear and power-law-like ways, respectively (figures 3*b* and 4*b*).

In summary, patterns of memory accuracy reflect patterns of word usages and social contact in humans. Specifically, memory performance is probably adapted to linear and power law relations in the environment. Along with related findings for other cognitive processes (e.g. reasoning, resource allocation, numerical representation [6–10]), the rational analyses of human memory and human environments support the notion that information-processing systems adaptively exploit environmental structures to solve information-processing problems.

### 1.3. The contribution of rational analysis

Our article makes three contributions to rational analysis and biology. First, we provide a methodological primer of how biologists can apply rational analysis to study animal cognition. Second, in so doing, we contribute to the comparative study of cognition across species, exploring parallels between humans and their closest living relatives, chimpanzees (*P. troglodytes*)*.* Third, we address the extent to which rational analysis, as a research methodology, hinges on arguments about evolution.

#### 1.3.1. Rational analysis for biologists

The rational analysis framework highlights the importance of exploring environmental pressures that shape cognition, which can provide valuable insights into the biology of animal behaviour and cognition. Cognitive ecology [29,30] is a subfield of biology that explores this problem. A classic example of this approach is the extensive work tying spatial memory performance to the foraging ecology of caching food. Species that scatter caches of food across a territory face the difficult challenge of remembering where they hid the food. Many researchers have argued that the pressure of remembering the location of caches should select for better spatial memory [31–33]. In fact, species that cache food more also have better spatial memory than species that cache less, even outside of the caching context [34–36].

Though cognitive ecology actively pursues the general question of how the environment shapes cognition, the rational analysis approach takes this a step further by specifying more precisely patterns that are observed in the environment to more strongly test the environment's influence on cognition. Rather than only stating that two species have different environments and should, therefore, have different cognition, rational analysis enables us to more precisely model how the environment shapes the cognition of species, and to what extent the cognitive system optimally solves the adaptive problems posed by the environment.

This modelling approach is rarely used in biology. An exception is a study by van der Vaart *et al*. [37], who developed a cognitive model of cache recovery behaviour. In the spirit of our opening example from robotics, they developed ‘virtual birds' that had to remember their caches. In doing so, the authors faced the problem of designing an artificial cognitive system that could encode, store and retrieve memories of cache locations. They used the quantitative insights of how frequency and recency of encountering objects in the world (food to cache) influence memory to retrieve those objects. Their model incorporated Anderson & Schooler's [4] memory functions derived from rational analysis to model how the memory system interacted with the environment to solve the problem of cache recovery. Though this used insights derived from rational analysis, it did not directly apply rational analysis to cognitive evolution. Here, we provide an application of rational analysis to biology.

#### 1.3.2. Comparative rational analysis

Cognitive scientists interested in investigating the role of the environment on cognition typically test this in humans by varying task environments [38,39]. Humans, however, are limited in the range of environments that they experience often enough to influence cognition. The comparative approach in biology, however, allows for another method for exploring the role of the environment on cognition. As different species face different environmental pressures, comparing across species allows biologists to compare whether different species with similar environments share aspects of behaviour and cognition [40–42].

The comparative approach can provide a powerful way to assess the utility of rational analysis. In discussing the usefulness of rational analysis for understanding the adaptive nature of memory, for instance, Shettleworth [41] lamented that we have few tests of how cognitive properties match properties of the environment in non-human species. Though van der Vaart *et al*. [37] used the insights of rational analysis to model animal memory, few have used the steps of rational analysis (figure 1) to better understand cognition (but see [43]). We leverage the comparative method to test the link between environment and cognition in another species—chimpanzees. This not only applies the rational analysis framework to a new species but also explores the evolutionary underpinnings of human cognition by comparing our environments and memory to those of our closest living relatives. Though humans demonstrate reliable patterns of both memory and the distribution of information across multiple social environments (word usage and social contact), it remains unclear whether these patterns are uniquely human or whether other species also demonstrate them.

Though one might expect that frequency and recency of encounters should predict future contact in a general sense, rational analysis predicts specifically that the same patterns observed in the environment will also be observed in the cognition. Therefore, for species with different social structures, frequency and recency of past contact may have different patterns predicting future contact. Rational analysis predicts that, whatever the pattern, it will be reflected in cognition. This allows biologists to leverage the diversity of social or other environments across species to make predictions about their cognitive evolution.

#### 1.3.3. Natural selection as a mechanism of rational analysis

Anderson & Schooler's [4] and Pachur *et al*.'s [24] work illustrates how much can be learned by exploring how the human cognitive system adapts to and exploits environmental structure to achieve key information-processing goals. There are, however, at least two mechanisms that can generate adaptive behaviour and cognition: learning and natural selection. If memory reflects the environment due to learning, memory mechanisms may change over an organism's lifetime due to environmental demands. In contrast, if natural selection is the mechanism generating an adaptive response to the environment, then, over evolutionary time, individuals that had memory mechanisms that reflected the environment would have more efficient memory performance, leading to better foraging success, enhanced mating opportunities and more positive social interactions. Anderson [1,44] proposed evolution by natural selection as a potential mechanism of rational analysis, but most work in this area has remained silent on the role of evolution.

Though others have embraced the Anderson & Schooler [4] approach to adaptive forgetting in non-human animals [37,45], some remain sceptical of its utility. Shettleworth [41], for example, questioned whether newspaper headlines reflect the environment in which our memory systems evolved. Shettleworth, therefore, challenged the evolutionary nature of rational analysis. Anderson & Schooler did not claim that the memory patterns are necessarily evolutionarily adapted, leaving open the possibility that they could be learned. But a cognitive ecology approach might hypothesize that memory systems are products of natural selection, and, therefore, the observed memory patterns may be evolutionary adaptations to the structure of ancestral environments. Indeed, the power law relationships found by Anderson & Schooler and Pachur *et al*. [24] are common in nature [46] and might well generalize from modern-day newspaper environments to ancestral (e.g. hunting, mating, social) environments. We contribute to this discussion by establishing to what extent the cognitive and environmental functions found in humans generalize to other species.

## 2. The rational analysis of chimpanzee memory

Here, we extend the rational analysis framework to a phylogenetically closely related species—the chimpanzee. Like Anderson & Schooler [4] investigated in humans, we are interested in chimpanzee memory as an information-processing problem. To begin the rational analysis, we establish the goals of the system (figure 1*a*). In our case, the goal of memory is to quickly retrieve memories relevant to the environmental situation at hand.

Second, we must measure the structure of the environment (figure 1*b*). We posit that encountering other individuals in one's group is an important environmental event for memory because it requires the retrieval of information about relationship status—including relatedness and dominance—with that individual, as well as that individual's specific past behaviours, such as previous cooperative interactions and mating advances or rejections. Previous encounters probably predict future encounters, and the memory system could leverage these patterns to predict what information should be retained for future use. Therefore, we measure patterns of social contact as a key aspect of the environment that may shape memory.

Third, we must assess the cognitive limitations on the system (figure 1*c*). Chimpanzees face constraints on storage space for memory, perhaps with stricter limitations than humans because they have about one third of the number of neurons that humans do [47]. With storage space at a premium, forgetting clears out room for quick retrieval of more relevant information.

Finally, we can integrate the previous three components to determine the optimal cognitive response (figure 1*d*) to achieve the goals of the memory system given the structure of the environment and cognitive constraints. For chimpanzee memory performance, we would predict that forgetting functions should reflect the probability of needing information in the environment. So, the relationship between forgetting and recency should mimic the relationship between social contact and recency. We, therefore, measure chimpanzee's forgetting functions.

Following Anderson & Schooler [4] and Pachur *et al*. [24], we analysed social contact patterns in wild chimpanzees and tested whether the frequency and recency of past within-group encounters predicted future contact. With the environmental patterns in hand, we then modelled chimpanzee memory performance to compare it to the structure of their social environment. Combined, these steps allow us to apply rational analysis to another species.

### 2.1. Chimpanzee social contact

#### 2.1.1. Environmental data

We used 19 years of data from the Kanyawara community of chimpanzees in Kibale National Park, Uganda from July 1988 to June 2006. During this time frame, the Kanyawara community ranged in an area about 38 km^2^ [48]. These chimpanzees were habituated to the presence of human observers without provisioning. The size and composition of the community remained relatively stable, averaging about 40–50 individuals (9–12 adult males and 12–15 adult females) during that time and totalling 143 chimpanzees over the course of the entire study period. Each day, two or more Ugandan field assistants located a party of chimpanzees by using nesting data from the previous day, listening for vocalizations or visiting recent feeding sites. The observers followed the party for as long as possible, typically until the animals built their night nests. If the party split, they followed the larger subgroup. At 15 min intervals, one observer used scan sampling [49] to record which chimpanzees were present in a party, regardless of age or sex. While such party-level follows may increase the number of observations of more social individuals, daily follows over several years ensure that all individuals were adequately sampled.

We converted the scan sample data into a matrix with all 10 153 possible pairings of the 143 chimpanzees as rows and all 4695 days of observation as columns. If an observer recorded a particular pair of chimpanzees as in the same group at the same time at least once for a particular day, we scored the pair as in contact for that day (*contact day*). If no observers scored the chimpanzees in the same group and either of the two chimpanzees was observed for at least 70% of the day, we scored the pair as not in contact for that day (*no contact day*). Otherwise, we scored the pair as ‘missing’ (NA) for that day. The 70% value provided a compromise between wanting to ensure that contacts were not missed and the resulting reduction in number of data points. We scored as missing (NA) all days in which a particular pair could not possibly interact because one member was not alive or was not in the community. About 90% of the 47-million cell matrix was scored as ‘missing’ and could not be used in this analysis. Despite the sparseness of the matrix, 4.9 million complete dyad observations remained for our analyses.

#### 2.1.2. Analysis

By analysing their environments, Anderson & Schooler [4] asked whether the pattern of word use over 100 days predicted the probability of use on day 101—much like a typical memory experiment that assesses the likelihood of recalling an item after *n* practice trials (e.g. [50]). Aggregating over items that appeared *n* times (e.g. 52) in a window of *m* days (e.g. 100) yields an empirical proportion (e.g. 52/100). This proportion served Anderson & Schooler as an estimate of the probability that an item used *n* times in *m* days will be needed on day *m* + 1. In addition to computing such need probabilities based on the frequency of prior encounter, Anderson & Schooler examined the predictive power of recency: they estimated the need probability of recall on day *m* + 1 as a function of how many days had passed since an item was last needed in a window of *m* days.

As Anderson & Schooler [4] did for humans, we assessed the probability of a chimpanzee needing to remember information about a conspecific in two ways. First, to estimate the need probability based on the frequency of past encounters, we used a moving window of 16 consecutive observation days with only one missing value allowed, resulting in 307 810 windows. We used a window of 16 days to balance the need between having large enough samples per frequency and having enough frequencies to detect patterns. We allowed a single missing value in the window to increase the number of windows we could analyse. For each 16 day window, we recorded the number of encounters for each pair of chimpanzees over the first 15 days (frequency) and the probability of contact on day 16. We report the mean (±binomial 95% CIs) values averaged over all chimpanzee pairs.

Second, to estimate the need probability based on the recency of past encounters, we computed the probability of contact for a pair based on the number of consecutive days (with the possibility of one missing value) since the last contact. Again, we report the mean (±binomial 95% CIs) values averaged over all chimpanzee pairs. We used a maximum recency of 15 days to match the frequency data. This resulted in 1.8 million recency events. Using binomially distributed maximum-likelihood estimation, we calculated the corrected Akaike Information Criterion (AIC*_c_*) values that correct for bias associated with small sample sizes [51] to test power and exponential functions with these data.

We analysed the data using R Statistical Software [52] v. 3.2.5 and packages *bbmle* [53], *car* [54], *Hmisc* [55], *lattice* [56] and *Rcpp* [57]. Data and R code are available in electronic supplementary materials and on the Dryad Data Repository (http://datadryad.org/) [58].

#### 2.1.3. Chimpanzee social contact patterns

At a broad level, the spacing patterns observed in chimpanzees mirror those observed in humans (figure 2*c*). In addition, figure 3*c* shows that the pattern of frequency of encounters over a 15 day period predicts future encounters among wild chimpanzees. The chimpanzees' social contact pattern exhibits regularities that match the human ones (figure 3*a,b*). Anderson & Schooler [4] reported a linear relationship between need probability and frequency. A linear regression demonstrates that future need probability is linearly related to past need probability also in chimpanzees (*p* = 0.14 + 0.05*n*; *R*^2^ = 0.95). Matching the human data, need probability strongly correlates with frequency of contact. If chimpanzee encounters are distributed at random, they would have a constant probability of contact, regardless of frequency (figure 3*c*).

Figure 4*c* shows the probability of encountering another chimpanzee as a function of the number of days that passed since the last encounter. Again, the chimpanzee results match the negatively accelerating function observed in the human data (figure 4*a,b*). The power function fits the data better than the exponential function (power AIC_c_: 84.2; exponential AIC_c_: 98.2; evidence ratio = 1 × 10^10^, suggesting that the power function has over 11 million times the weight of evidence relative to the exponential function). This finding matches the power function reported for humans by Anderson & Schooler [4] and Pachur *et al*. [24]. Moreover, if the chimpanzees moved around their territory at random, this should produce an exponential pattern of encounters with recency [59]. Our power-law data, therefore, reflect structure in chimpanzee social contact.

### 2.2. Chimpanzee memory

**Figure 5.**
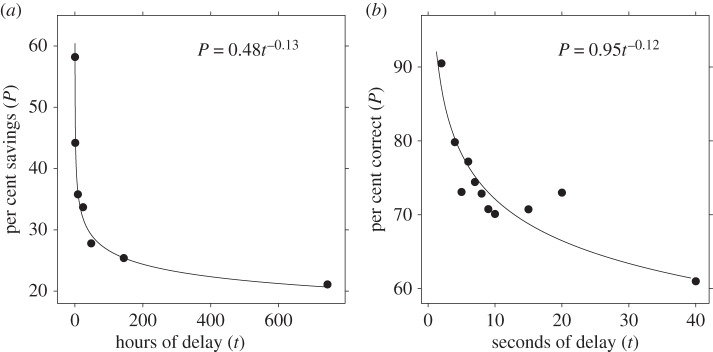
(*a*) Ebbinghaus's [17] classic forgetting function in humans is based on retention assessed as per cent savings in relearning a list of nonsense syllables. (*b*) The chimpanzee forgetting function is based on two-sample delayed matching-to-sample tasks [60–62]. Curves illustrate best fitting power functions.

Anderson & Schooler [4] used cognitive data (figure 5*a*) to inform predictions about patterns in the human environment. Schooler & Anderson [5] used statistical patterns in the environment to make novel behavioural predictions. Similarly, our chimpanzee social environment data allow us to reverse the direction and make predictions about chimpanzee cognition. The power-law pattern observed in social contact data (figure 4*c*) provides a critical regularity of the environment in which chimpanzee memory must perform. Based on these data, we predicted that chimpanzee memory retention would also follow a power law, as a response to the pattern in the social environment.

To test this prediction, we combined data from chimpanzee delayed matching-to-sample tasks across three prior studies [60–62]. These tasks presented a ‘sample’ object to remember, hid the sample from view for a fixed period, and then presented the sample and a distractor object. If the chimpanzee chose the sample object, it received a food reward; otherwise, it received nothing. Experimenters varied the retention interval within chimpanzees, allowing us to model memory functions. Though these data have been available for decades, no one has fitted power and exponential functions to the data to test which function best accounts for their forgetting patterns. We found that, like the chimpanzee social contact data, the power function fits the chimpanzee memory data better than the exponential function (power AICc: 53.9; exponential AICc: 62.3; evidence ratio = 54.1; figure 5*b*).

These data indicate that chimpanzee memory makes a similar bet as human memory: namely, the more time that has passed since encountering a stimulus, the more likely it is that the stimulus will not need to be recalled in the future, and memories of such stimuli can be forgotten. Thus, the statistical regularities carry over from chimpanzee social environments to their cognition, as predicted by rational analysis.

## 3. Discussion

Field data on chimpanzee group composition demonstrate that the frequency and recency of their social contact predict future contact in a reliable way, with linear and power functions, respectively. Experimental data on chimpanzee memory indicate that their probability of forgetting depends directly on the recency with which they encountered the memory stimuli. Critically, the forgetting function shows the same relationship with recency as social contact patterns, namely a power-law decrease with recency. Combined, these results provide (i) a key case study of the application of rational analysis to biological systems, (ii) an opportunity to employ rational analysis to better understand comparative aspects of human and non-human cognition, and (iii) a chance to explore natural selection as a mechanism relevant for rational analysis.

### 3.1. Applying rational analysis

Psychologist Walter Mischel once related that ‘psychologists treat our theories like toothbrushes; no self-respecting person wants to use anyone else's’ [63, p. 444]. We agree, and would like to add that this is probably also true for cross-disciplinary research: too infrequently do research methodologies and theories developed in one field or discipline carry over into neighbouring ones. This article makes such an attempt to bridge disciplinary boundaries.

The rational analysis framework posits that cognitive processes are adapted to solve problems that the environment imposes on agents. Though animal researchers have incorporated the fruits of rational analysis labours [37], few have directly applied the rational analysis approach to understand the evolution of cognition in other species. We stepped through the components of rational analysis (figure 1) to ask to what extent chimpanzee memory performance matches the probability of needing information in a social environment. Field and experimental data show that chimpanzee memory performance follows the same statistical pattern—a power function—as chimpanzee social contact.

Rational analysis does not always predict linear and power-law patterns, however. The environment acts as a constraint on the cognitive system, so different species (or even populations of the same species) may face different patterns in their environment. For social contact, some species have more cohesive or less structured social organization. The cohesive group composition of baboons, for instance, would not result in the same power-law pattern of recency observed in chimpanzees because baboon group members are in constant contact. Likewise, less structured groups such as large flocks of birds or swarms of insects may not show this pattern because previous contact simply does not predict future contact. Different social organizations result in different patterns of contact.

In addition to social organization, other factors impose information-processing demands. Caching species such as Clark's nutcrackers (*Nucifraga columbiana*) face strong selective pressures to remember where they have hidden seeds in the winter [64]. The seasons impose tight schedules on how often these birds need to recall the locations of their food deposits. But these birds have few social interactions, so their memory systems may be tuned more for the statistical patterns of cache recovery rather than social contact. Thus, we must characterize the statistical structure of each species individually before predicting its effect on cognition.

Our work answers the call of Shettleworth [41], who lamented the lack of applications of rational analysis to other species. We offer a ‘proof-of-concept’ that rational analysis can be a valuable tool used in biology to better understand cognition and behaviour in other species. Carefully measuring regularities in the physical and social environment can provide insights into optimal solutions to a range of problems faced by animals, including foraging, mating, predator avoidance and navigation.

### 3.2. Rational analysis in humans and chimpanzees

Systematically exploring parallels and differences between our and other hominid's information processing can offer key insights into the evolution of hominid cognition [65–67]. We find that the chimpanzee social environment and memory performance exhibit similar statistical regularities as those found in humans. There are at least two possible explanations for the origin of this similarity. First, both species share fission–fusion social dynamics [68]. Chimpanzees live in fission–fusion societies in which members of ‘communities' of about 45 individuals (but sometimes more than 150 individuals [69]) travel in fluid subgroups that change in size and composition over the course of hours or days [70]. Humans show similarly fluid social dynamics [68]. With the dynamic nature of fission–fusion systems, recent and frequent contact with social partners predicts that future contact is likely. Whereas, when a group fission occurs, subgroups may travel to different parts of the range, thereby reducing the probability of future contact. The fission–fusion dynamics exhibited by chimpanzees and humans may provide a unique adaptive pressure on cognition because group members are encountered after varying intervals. Further modelling efforts are needed to determine whether the fission–fusion dynamics uniquely generate the contact patterns observed here.

An alternative explanation is that humans and chimpanzees share these statistical regularities not because they share fission–fusion dynamics but because they share an evolutionary history independent of these dynamics. That is, the linear and power-law patterns of frequency and recency on future contact may simply be phylogenetic constraints. To fully test between these two possibilities, we must conduct similar rational analyses for other species that vary in their social structure. Regardless of whether these patterns are adaptations to fission–fusion dynamics or are phylogenetic constraints, the fact that humans and chimpanzees share these patterns hints at a shared social environment that has critical implications for cognitive evolution.

### 3.3. Mechanisms of rational analysis

A key open question raised by Anderson & Schooler's [4] findings is whether the match of memory and the environment results from memory adapting to an individual's environment over its lifetime (learning) or from memory adapting to a population's environment over evolutionary time (natural selection). Shettleworth [41] was sceptical that Anderson & Schooler's patterns of word usage in newspaper headlines reflected a general property of human environments that were present during the evolution of human memory. Our data speak to this question.

The chimpanzees included in our social contact pattern dataset lived in natural conditions and exhibited natural social contact patterns. The chimpanzees in our memory data, however, lived in captivity. Captive chimpanzees typically live in constant contact with their group mates, which would generate different patterns of social contact; recency and frequency would not predict future contact in a fixed group. The memory data, therefore, was probably not generated by a mechanism that learned patterns of social contact over the lifetime of the chimpanzee, because these patterns of contact will not follow a power law for captive chimpanzees. The fact that memory performance in captive chimpanzees reflects social contact in wild chimpanzees provide evidence to cognitive scientists that natural selection can act as the mechanism that matches the properties of cognition to the properties of the environment as suggested by rational analysis.

### 3.4. Rational analysis of cognitive and behavioural evolution

Rational analysis parallels the fields of cognitive ecology [29,30] and evolutionary psychology [71] by emphasizing the tight link between the environment and cognition. It goes beyond these fields; however, by more explicitly measuring the statistical structure of the environment to predict what kind of cognitive system would best solve the problems faced by that environment. Therefore, this approach treats many aspects of cognition as adaptations to environmental problems rather than just constraints. Biologists, for example, often consider memory to be a constraint on the cognitive system due to limited storage capacity [25,72]. Rational analysis maintains that—though constraints on capacity exist—memory is an adaptation with properties that solve the adaptive problem of retrieving information relevant to the situation at hand.

But rational analysis extends beyond memory. Patterns of social contact are relevant to a number of key biological problems, including the evolution of cooperation, cultural transmission and disease transmission. Quantitatively assessing aspects of the social environment, such as contact patterns, can provide critical insights into these issues. Existing models of cooperation, for example, tend to ignore such contact patterns, though they may incorporate broader-scale social networks. Yet, the pattern of contact probably has important implications for models of cooperation such as reciprocity; frequency and recency of interactions, combined with memory constraints, may determine what types of reciprocal strategies can evolve [24,25,73]. Frequent and recent encounters, for instance, aid remembering past defections. But spaced-out encounters aid forgetting and, therefore, forgiving mistaken defections. The beneficial and detrimental roles of memory will interact with social contact to influence the evolution of cooperation. Furthermore, contact patterns directly shape transmission of both cultural information and disease. Frequency and recency of contact regulate the magnitude and timing of exposure to memes and pathogens, thereby determining the extent and rate of their spread [74–76]. Thus, these patterns of contact provide metrics of the social environment that influence key cognitive and disease-based factors critical to behavioural evolution.

## 4. Conclusion

Herbert Simon once remarked that ‘Human rational behavior … is shaped by a scissors whose two blades are the structure of task environments and the computational capabilities of the actor’ [77, p. 7]. Though Simon focused on the current environment, his notion also applies to the evolutionary environment. Our findings hint that human memory may have evolved as an adaptation that is functional with respect to the statistical properties of the environment. The rational analysis framework allows us to understand how the statistical properties of our and other species' environments align and differ, so that we can fully grasp in what respects human information-processing capacities are unique and in what respect they are shared with other species. It is perhaps only once we have examined how the statistical properties of human environments and memory performance differ from and resemble those of other species that we may grasp how adaptive human and other species' cognitive capacities really are.

## Supplementary Material

stevens_data

## Supplementary Material

Chimpanzee social contact data—Matrix of contact data for 143 chimpanzees over 4,695 days Code—R script and C++ code used to analyze data and generate figures

## Data accessibility

Data and R code are available in electronic supplementary materials and on the Dryad Data Repository: http://dx.doi.org/10.5061/dryad.bh330 [58].
