# Supplementary material for: Reflections of the social environment in chimpanzee memory: applying rational analysis beyond humans
Source: R Soc Open Sci. 2016 Aug 3;3(8):160293. doi: 10.1098/rsos.160293 (PMC5108956; doi:10.1098/rsos.160293)
Supplement: Chimpanzee social contact data—Matrix of contact data for 143 chimpanzees over 4,695 days Code—R script and C++ code used to analyze data and generate figures [file rsos160293supp2.pdf]

ReadMe file
Created on 8 Jun 2016 by Jeffrey R. Stevens (jeffrey.r.stevens@gmail.com)

*********************************************************
If you use the data, please cite the following:
Stevens, J.R., Marewski, J.N., Schooler, L.J., and Gilby, I.C. (2016). Reflections of the social environment in chimpanzee memory: applying rational analysis beyond humans. Royal Society Open Science.
*********************************************************

Summary: These data were extracted from 19 years of data from the Kanyawara community of chimpanzees in Kibale National Park, Uganda from July 1988 to June 2006. The data rows are dyads of chimpanzees and columns are days. Cell values represent whether the dyad was observed together (1) or not (0) on that day. If neither chimpanzee was observed for more than 70% of the day or if the chimpanzees could not possibly have been in contact (e.g., at least one was not living), the values are blank.

Data files:
- stevens_data.csv
  - column 1 - ID for chimpanzee #1
  - column 2 - ID for chimpanzee #2
  - column 3 - sex of chimpanzee #1 (1 = male, 2 = female)
  - column 4 - sex of chimpanzee #2 (1 = male, 2 = female)
  - column 5 - flag for whether chimpanzee #1 is mother of chimpanzee #2 (1 = yes, 0 = no)
  - column 6 - flag for whether chimpanzee #2 is mother of chimpanzee #1 (1 = yes, 0 = no)
  - columns 7-4701 - cooccurrence matrix for each of 4,695 days of observation (columns) for each dyad (rows) (1 = observed together, 0 = not observed together, blank = missing data)

R code:
- stevens_etal_rcode.R--code for processing data and generating figures

C++ code:
- stevens_etal_frequency.cpp--code for calculating frequency analysis (needed to run R code)
- stevens_etal_recency.cpp--code for calculating recency analysis (needed to run R code)

```r
###################################################
### stevens_contact_rcode.R
### Created by Jeffrey R. Stevens on 11 Mar 2010 (jeffrey.r.stevens@gmail.com),
###  finalized on 8 Jun 2016
### Summary: This script analyzes and produces plots for spacing, frequency, and
###   recency data from chimpanzee social contact data.
### Instructions: Place this file, the data file, and the cpp files in a directory.
###   Set the R working directory to this directory. At the R command prompt, type
###   > source("stevens_rcode.R")
###   This will run the script, adding all of the calculated variables to the
###   workspace and saving figures.
### Uses: This script can be reproduced and modified for personal and scientific use.
###   Please cite the published article when publishing anything based on this script.
### Data files:
###  stevens_data.csv
###   column 1 - ID for chimpanzee subject #1
###   column 2 - ID for chimpanzee subject #2
###   column 3 - sex of chimpanzee 1
###   column 4 - sex of chimpanzee 2
###   column 5 - flag for whether chimpanzee 1 is mother of chimpanzee 2 (1 = yes, 0 = no)
###   column 6 - flag for whether chimpanzee 2 is mother of chimpanzee 1 (1 = yes, 0 = no)
###   columns 7-4701 - cooccurrence matrix for each of 4,695 days of observation (columns) for each dyad (rows) (1 = observed together, 0 = not observed
together, blank = missing data)
###################################################

###################################################
### Load libraries and C++ files
###################################################

rm(list=ls())                 # clears all variables

library(bbmle, quietly = T)               # required for mle2 to calculate maximum likelihood estimates
library(car)                              # required for Recode
library(Hmisc)                            # required for binconf to calculate binary confidence intervals
library(lattice)                          # required for lattice graphs
library(Rcpp)                             # required to run C++ scripts
sourceCpp("stevens_etal_frequency.cpp")   # C++ script needed for findFrequency()
sourceCpp("stevens_etal_recency.cpp")     # C++ script needed for findRecency()

col.blind <- c("#0072B2", "#E69F00", "#009E73", "#D55E00")  # prepare color-blind-safe colors

###################################################
### Spacing plots
###################################################
###################
## New York Times spacing data (Anderson & Schooler 1991)
###################
# Input and process data
nytimes_spacing <- data.frame(days = c(6, 7, 8, 9, 10, 14, 26, 31, 85, 93, 98, 100, 100, 103, 105, 106, 107, 108, 30, 32, 35, 37, 40, 46, 100, 20, 96,
105, 110, 25, 2, 4, 6, 6, 8, 10, 11, 16, 17, 18, 22, 22, 23, 25, 28, 30, 31, 35, 36, 37, 37, 41, 42, 43, 46, 49, 51, 52, 53, 54, 57, 60, 62, 65, 67, 69,
71, 73, 75, 76, 77, 78, 79, 81, 83, 84, 86, 88, 91, 94, 98, 99, 100, 103, 104, 105, 106, 108, 110, 111, 114, 115, 117, 118, 1, 2, 2, 4, 6, 7, 7, 10, 11,
12, 13, 13, 15, 18, 18, 20, 21, 21, 23, 24, 25, 26, 26, 28, 30, 31, 32, 33, 34, 35, 36, 37, 38, 39, 40, 41, 42, 44, 44, 45, 46, 47, 49, 50, 52, 52, 53,
55, 56, 57, 58, 58, 59, 60, 61, 62, 63, 64, 65, 66, 67, 68, 69, 70, 71, 72, 73, 74, 75, 76, 78, 79, 80, 81, 82, 83, 84, 85, 86, 87, 88, 89, 90, 91, 92,
93, 94, 95, 96, 98, 99, 100, 101, 104, 107, 108, 109, 110, 111, 113, 114, 115, 116, 117, 118, 119), wordnum = c(1, 1, 1, 1, 1, 1, 1, 1, 1, 1, 1, 1, 1, 1,
1, 1, 1, 1, 2, 2, 2, 2, 2, 2, 2, 3, 3, 3, 3, 4, 5, 5, 5, 5, 5, 5, 5, 5, 5, 5, 5, 5, 5, 5, 5, 5, 5, 5, 5, 5, 5, 5, 5, 5, 5, 5, 5, 5, 5, 5, 5, 5, 5, 5, 5,
5, 5, 5, 5, 5, 5, 5, 5, 5, 5, 5, 5, 5, 5, 5, 5, 5, 5, 5, 5, 5, 5, 5, 5, 5, 5, 5, 5, 5, 6, 6, 6, 6, 6, 6, 6, 6, 6, 6, 6, 6, 6, 6, 6, 6, 6, 6, 6, 6, 6, 6,
6, 6, 6, 6, 6, 6, 6, 6, 6, 6, 6, 6, 6, 6, 6, 6, 6, 6, 6, 6, 6, 6, 6, 6, 6, 6, 6, 6, 6, 6, 6, 6, 6, 6, 6, 6, 6, 6, 6, 6, 6, 6, 6, 6, 6, 6, 6, 6, 6, 6, 6,
6, 6, 6, 6, 6, 6, 6, 6, 6, 6, 6, 6, 6, 6, 6, 6, 6, 6, 6, 6, 6, 6, 6, 6, 6, 6, 6, 6, 6, 6, 6, 6, 6))
nytimes_spacing$word <- Recode(nytimes_spacing$wordnum, "1='Qaddafi'; 2='Challenger'; 3='American'; 4='North'; 5='Reagan'; 6='of'") # recode numbers to
words
nytimes_spacing$word <- factor(nytimes_spacing$word,levels(factor(nytimes_spacing$word))[c(5, 2, 1, 3, 6, 4)])                      # reorder words
```

```r
# Generate NY Times spacing plot
nytimes_spacing_plot <- dotplot(word ~ days, data = nytimes_spacing, aspect = 1,                                          # plot data
  xlab = "Day", ylab = "Word",
  par.settings = list(axis.text = list(cex = 2.5), par.xlab.text = list(cex = 3), par.ylab.text = list(cex = 3)),
  panel = function(x, y, ...) {
    panel.dotplot(x, y,  pch = 20, col = "black", cex = 1.25)
    panel.text(x = 0, y = 6.35, labels = "(a)", cex = 2.5)  # add subfigure label
  }
)

###################
## Human social contact spacing data (Pachur et al. 2014)
###################
# Input and process data
pair1 <- c(1, 1, 1, 1, 1, 1, 1, 1, 1, 1, 1, 1, 1, 1, 1, 1, 1, 1, 1, 1, 1, 1, 1, 1, 1, 1, 1, 1, 1, 1, 1, 1, 1, 1, 1, 1, 1, 1, 1, 1, 1, 1, 1, 1, 1, 1, 1,
1, 1, 1, 1, 1, 1, 1, 1, 1, 1, 1, 1, 1, 1, 1, 1, 1, 1, 1, 1, 1, 1, 1, 1, 1, 1, 1, 1, 1, 1, 1, 1, 1, 1, 1, 1, 1, 1, 1, 1, 1, 1, 1, 1, 1, 1, 0, 0, 1, 1, 1)
pair2 <- c(1, 0, 0, 1, 1, 1, 0, 0, 0, 0, 0, 1, 1, 1, 0, 0, 0, 1, 0, 1, 1, 1, 1, 1, 1, 1, 1, 1, 0, 0, 1, 1, 0, 0, 1, 1, 0, 1, 1, 1, 1, 0, 1, 1, 1, 1, 1,
1, 1, 1, 1, 0, 1, 1, 1, 0, 1, 1, 1, 1, 1, 1, 1, 1, 1, 1, 1, 1, 1, 0, 1, 1, 1, 0, 1, 1, 1, 1, 1, 0, 1, 0, 1, 1, 0, 1, 0, 0, 0, 0, 0, 1, 1, 0, 0, 1, 0, 0)
pair3 <- c(0, 0, 0, 0, 0, 0, 0, 0, 0, 0, 0, 0, 0, 0, 0, 0, 0, 0, 0, 0, 0, 0, 0, 0, 0, 0, 0, 0, 0, 0, 0, 0, 0, 0, 0, 0, 0, 0, 0, 0, 0, 0, 0, 0, 0, 0, 0,
0, 0, 0, 0, 0, 0, 0, 0, 0, 0, 0, 0, 0, 0, 0, 0, 1, 0, 0, 0, 0, 0, 0, 0, 0, 0, 0, 0, 0, 0, 0, 0, 0, 0, 0, 0, 0, 0, 0, 0, 0, 0, 0, 0, 0, 0, 0, 0, 0, 0, 0)
pair4 <- c(0, 0, 0, 0, 0, 0, 0, 0, 0, 0, 0, 0, 0, 0, 0, 0, 0, 0, 0, 0, 0, 0, 0, 0, 0, 0, 0, 0, 0, 0, 0, 0, 0, 0, 0, 0, 0, 0, 0, 0, 0, 0, 0, 0, 0, 0, 0,
0, 0, 0, 0, 0, 0, 0, 0, 0, 0, 0, 0, 0, 0, 0, 0, 0, 0, 0, 0, 1, 0, 0, 0, 0, 0, 0, 1, 0, 0, 0, 0, 0, 0, 1, 0, 0, 0, 0, 0, 0, 0, 0, 0, 0, 0, 0, 0, 0, 0, 0)
pair5 <- c(0, 0, 0, 0, 0, 0, 0, 0, 0, 0, 0, 0, 0, 0, 0, 0, 0, 0, 0, 0, 0, 0, 0, 0, 0, 0, 0, 0, 0, 0, 0, 0, 0, 0, 0, 0, 0, 0, 0, 0, 0, 0, 0, 0, 0, 0, 1,
0, 0, 1, 1, 1, 1, 1, 0, 0, 1, 1, 1, 1, 1, 0, 0, 1, 1, 1, 1, 1, 0, 0, 1, 1, 1, 0, 0, 0, 0, 0, 1, 0, 0, 0, 0, 0, 0, 0, 0, 0, 0, 0, 0, 0, 0, 0, 0, 0, 0, 0)
pair6 <- c(0, 0, 0, 0, 0, 0, 0, 0, 0, 0, 0, 0, 0, 1, 1, 1, 1, 1, 1, 1, 1, 1, 1, 1, 1, 1, 1, 1, 0, 0, 0, 0, 0, 0, 0, 0, 0, 0, 0, 0, 0, 0, 0, 0, 0, 0, 0,
0, 1, 0, 0, 0, 0, 0, 0, 1, 0, 0, 0, 0, 0, 0, 0, 0, 0, 0, 0, 0, 0, 1, 0, 0, 0, 0, 0, 0, 0, 0, 0, 0, 0, 0, 0, 0, 0, 0, 0, 0, 0, 0, 0, 0, 0, 0, 0, 0, 0, 0)
allpairs <- c(pair1, pair2, pair3, pair4, pair5, pair6) # create vector of pair encounters
pairs <- c(rep("A-B", 98), rep("A-C", 98), rep("A-D", 98), rep("A-E", 98), rep("A-F", 98), rep("A-G", 98))           # create vector of pair labels
human_social_spacing <- data.frame(allpairs, pairs)                                                                    # create data frame of pair labels
and encounters
human_social_spacing$pairs <- factor(human_social_spacing$pairs, levels(factor(human_social_spacing$pairs))[c(6:1)])  # reorder pair labels
human_social_spacing$day <- rep(1:98, 6)                                                                               # create vector of days
human_social_spacing$contact <- ifelse(human_social_spacing$allpairs == 1, human_social_spacing$day, NA)               # create vector of contacts with NA
for no contact (to plot only 1s)

# Human social contact spacing plot
human_social_spacing_plot <- dotplot(pairs ~ contact, data = human_social_spacing, aspect = 1, # plot data
  xlab = "Day", ylab = "Human contact partners",
  par.settings = list(axis.text = list(cex = 2.5), par.xlab.text = list(cex = 3), par.ylab.text = list(cex = 3)),
  panel = function(x, y, ...) {
    panel.dotplot(x, y,  pch = 20, col = "black", cex = 1.25)
    panel.text(x = 0.2, y = 6.35, labels = "(b)", cex = 2.5)  # add subfigure label
  }
)

###################
## Chimpanzee spacing data
###################
# Input and process data
pair1 <- c(1, 1, 1, 1, 1, 1, 1, 1, 1, 1, 1, 1, 1, 1, 1, 1, 1, 1, 1, 1, 1, 1, 1, 1, 1, 1, 1, 1, 1, 1) # 6, 111 (Slim-Johnny)
pair2 <- c(1, 1, 1, 1, 1, 0, 0, 0, 1, 1, 1, 1, 1, 1, 1, 1, 1, 1, 1, 1, 1, 0, 1, 1, 1, 1, 1, 1, 1, 1) # 103, 111 (Slim-Pepsi)
pair3 <- c(0, 0, 0, 0, 0, 0, 0, 0, 0, 0, 0, 0, 0, 0, 0, 0, 0, 0, 0, 0, 0, 0, 0, 0, 0, 0, 0, 0, 0, 1) # 79, 111 (Slim-Temba)
pair4 <- c(0, 0, 0, 0, 0, 0, 0, 0, 0, 0, 0, 0, 0, 0, 0, 0, 0, 0, 0, 0, 0, 0, 1, 1, 0, 0, 0, 1, 1, 0) # 49, 111 (Slim-Indiana)
pair5 <- c(0, 0, 0, 0, 0, 0, 0, 0, 0, 0, 0, 0, 0, 0, 0, 0, 0, 0, 0, 0, 0, 1, 1, 1, 1, 1, 0, 0, 0, 0) # 28, 111 (Slim-Ekisigi)
pair6 <- c(0, 0, 1, 1, 0, 0, 0, 0, 0, 0, 0, 1, 0, 1, 1, 1, 0, 0, 0, 0, 0, 0, 0, 0, 1, 1, 1, 0, 0, 1) # 22, 111 (Slim-Bono)
allpairs <- c(pair1, pair2, pair3, pair4, pair5, pair6) # create vector of pair encounters
pairs <- c(rep("Slim-Johnny", 30), rep("Slim-Pepsi", 30), rep("Slim-Temba", 30), rep("Slim-Indiana", 30), rep("Slim-Ekisigi", 30), rep("Slim-Bono", 30))
# create vector of pair labels
chimp_spacing <- data.frame(allpairs, pairs)                                            # create data frame of pair labels and encounters
chimp_spacing$pairs <- factor(chimp_spacing$pairs, levels(chimp_spacing$pairs)[c(1:3, 6, 5, 4)])  # reorder pair labels
```

```
chimp_spacing$day <- rep(1:30, 6)                                                          # create vector of days
chimp_spacing$contact <- ifelse(chimp_spacing$allpairs == 1, chimp_spacing$day, NA)         # create vector of contacts with NA for no contact (to
plot only 1s)

# Generate chimpanzee spacing plot
chimp_spacing_plot <-   dotplot(pairs ~ contact, data = chimp_spacing, aspect = 1,  # plot data
  xlab = "Day", ylab = "Chimpanzee pair",
  par.settings = list(axis.text = list(cex = 2.5), par.xlab.text = list(cex = 3), par.ylab.text = list(cex = 3)),
  panel = function(x, y, ...) {
    panel.dotplot(x, y,  pch = 20, col = "black", cex = 1.25)
    panel.text(x = 0.7, y = 6.35, labels = "(c)", cex = 2.5)  # add subfigure label
  }
)

## Print all spacing plots
png(filename = "spacing.png", width = 1800, height = 500)           # create png file
print(nytimes_spacing_plot, split = c(1, 1, 3, 1), more = TRUE)     # print human NY Times headline data
print(human_social_spacing_plot, split = c(2, 1, 3, 1), more = TRUE)# print human contact data
print(chimp_spacing_plot, split = c(3, 1, 3, 1), more = FALSE)      # print chimpanzee contact data
dev.off()

###################################################
### Frequency analysis
###################################################
###################
## NY Times headlines frequency data (Anderson & Schooler 1991)
###################
# Input and process data
nytimes_freq <- round(c(1.19, 4.37, 4.77, 10.33, 13.31, 17.89, 28.22, 33, 38.37, 43.14, 48.31, 55.66, 65.81, 85.88, 95.62), 0) # frequency data
nytimes_prob <- c(0.006, 0.028, 0.044, 0.079, 0.1099, 0.165, 0.239, 0.305, 0.385, 0.408, 0.4896, 0.541, 0.6787, 0.822, 0.925)  # probability data
nytimes_reg <- lm(nytimes_prob ~ nytimes_freq)  # calculate linear regression

# Generate NY Times headlines frequency plot
nytimes_freq_plot <- xyplot(nytimes_prob ~ nytimes_freq, aspect = 1,  # plot data
  xlab = expression(paste("Frequency of word in past 100 days (", italic("n"), ")")),
  ylab = expression(paste("Probability of word on day 101 (", italic("P"), ")")), ylim = c(-0.05, 1.05),
  par.settings = list(axis.text = list(cex = 2.25), par.xlab.text = list(cex = 2.75), par.ylab.text = list(cex = 2.75)),
  panel = function(x, y, ...) {
    panel.xyplot(x, y, type = "b", pch = 20, col = "black", lwd = 3, cex = 2)
    panel.abline(reg = nytimes_reg, col = "black")                                                 # plot regression line
    panel.text(x = 1, y = 1, labels = "(a)", cex = 3.5)                                            # include subfigure letter
    panel.text(35, 0.9, label = expression(paste(italic("P"), " = -0.01+0.01", italic("n"))), cex = 2.75) # include linear regression info
    panel.text(25, 0.82, label = expression(paste(italic("R")^2," = 0.99")), cex = 2.75)                  # include R-squared value
  }
)

###################
## Human social frequency data (Pachur et al. 2014)
###################
# Input and process data
human_social_freq <- c(0.032876624, 0.034385551, 0.06103424, 0.084376752, 0.121121972, 0.154643059, 0.175348496, 0.20713073, 0.246972318, 0.274725275,
0.305415617, 0.32746197, 0.364150943, 0.420374707, 0.450797872, 0.452095808, 0.517786561, 0.541019956, 0.538461538, 0.583815029, 0.601973684,
0.650980392, 0.714876033, 0.756097561, 0.72, 0.827380952, 0.818897638, 0.871165644, 0.936170213, 0.951672862, 0.987951807)  # probability data
days0to30 <- 0:30                                   # create vector of frequencies
human_freq_reg <- lm(human_social_freq ~ days0to30) # calculate linear regression

# Generate human social contact frequency plot
human_social_freq_plot <- xyplot(human_social_freq ~ days0to30, aspect = 1,   # plot data
  xlab = expression(paste("Frequency of contact in last 30 days (", italic("n"), ")")),
  ylab = expression(paste("Probability of contact on day 31 (", italic("P"), ")")),
  par.settings = list(axis.text = list(cex = 2.25), par.xlab.text = list(cex = 2.75), par.ylab.text = list(cex = 2.75)),
```

```
  panel = function(x, y, ...) {
    panel.xyplot(x, y, type = "b", pch = 20, col = "black", lwd = 3, cex = 2)
    panel.abline(reg = human_freq_reg, col = "black")                                         # plot regression line
    panel.text(x = -0.2, y = 1, labels = "(b)", cex = 3.5)                                    # include subfigure letter
    panel.text(10, 0.9, label = expression(paste(italic("P"), " = -0.01+0.03", italic("n"))), cex = 2.75) # include linear regression info
    panel.text(7, 0.82, label = expression(paste(italic("R")^2," = 0.99")), cex = 2.75)                   # include R-squared value
  }
)

###################
## Chimpanzee frequency data
###################
# Read in observed data
cooccur <- read.csv("stevens_data.csv")                    # read in observed cooccurrence matrix
observed_matrix <- as.matrix(cooccur[, -c(1:6)])          # remove first six demographic columns and convert to matrix
mean_contact_prob <- mean(observed_matrix, na.rm = TRUE)  # calculate overall mean contact probability

# Initiate variables
n <- 143                 # assign population size
days <- 4695             # assign number of days of observation
total_cells <- n * days # calculate total number of matrix cells
window_size <- 15        # assign window size
nas_allowed <- 1         # assign number of NAs allowed
freq <- 0:window_size    # create vector of frequencies

# Calculate probability of contact on day 16 given frequencies of 0-15 days of contact
observed_freq_all <- findFrequency(observed_matrix, window_size, nas_allowed)             # run frequency analysis from C++ script
observed_freq_contacts <- observed_freq_all$freq_contacts[1:(window_size + 1)]            # extract number contacts at different frequencies
observed_freq_observations <- observed_freq_all$freq_observations[1:(window_size + 1)]    # extract total number of observations at different frequencies
num_freq_windows <- sum(observed_freq_observations)                                        # calculate total number of windows used in frequency analysis
observed_freq <- data.frame(binconf(observed_freq_contacts, observed_freq_observations))  # calculate probability of contact and confidence intervals for
binomial probabilities
observed_freq$freq <- freq                                                                 # include frequencies in data frame
names(observed_freq) <- c("pcontact", "lower", "upper", "freq")                           # rename columns

# Calculate linear regression and coefficients
freq_reg <- summary(lm(observed_freq$pcontact ~ freq))  # calculate regression
w1 <- round(coef(freq_reg)[1], 2)                       # extract intercept
w2 <- round(coef(freq_reg)[2], 2)                       # extract slope
freq_r2 <- round(freq_reg$adj.r.squared, 2)             # extract adjusted R^2

# Generate chimpanzee frequency plot
chimp_freq_plot <- xYplot(Cbind(pcontact, lower, upper) ~ freq, data = observed_freq, # plot data
  aspect = 1, type = "b", cex = 2, pch = 20, col = "black", lwd = 3,
  ylim = c(-0.05, 1.05),
  xlab = expression(paste("Frequency of contact in last 15 days (", italic("n"), ")")),
  ylab = expression(paste("Probability of contact on day 16 (", italic("P"), ")")),
  par.settings = list(axis.text = list(cex = 2.25), par.xlab.text = list(cex = 2.75), par.ylab.text = list(cex = 2.75)),
  offset = unit(0.25,'inches'),
  panel = function(...) {
    panel.xYplot(...)
    panel.text(x = 0, y = 1, labels = "(c)", cex = 3.5)   # include subfigure letter
    panel.abline(reg = freq_reg, col = "black")           # plot regression line
    panel.abline(h = mean_contact_prob, lty = 2)          # plot regression line
    panel.text(4.8, 0.9, label = substitute(paste(italic("P"), " = ", w1, "+", w2, italic("n")), env = list(w1 = w1, w2 = w2)), cex = 2.75) # include
linear regression info
    panel.text(3.4, 0.82, label = substitute(paste(italic("R")^2," = ", freq_r2),env = list(freq_r2 = freq_r2)), cex = 2.75)                # include R-
squared value
  }
)
```

```
## Print all frequency plots
png(filename = "frequency.png", width = 2000, height = 700)         # create PNG file
print(nytimes_freq_plot, split = c(1, 1, 3, 1), more = TRUE)        # plot human NY Times headline data
print(human_social_freq_plot, split = c(2, 1, 3, 1), more = TRUE)   # plot human social contact data
print(chimp_freq_plot, split = c(3, 1, 3, 1), more = FALSE)         # plot chimpanzee social contact data
dev.off()                                                           # close PNG file

###################################################
### Recency analysis
###################################################
###################
## NY Times headlines recency data (Anderson & Schooler 1991)
###################
# Input and process data
nytimes_rec <- c(1.23, 2.02, 3.20, 3.97, 5.10, 6.05, 6.80, 7.75, 9.25, 10.19, 13, 18.05, 22.90, 28.32, 33.36, 38.21, 43.07, 48.1, 55.76, 66.02, 75.92,
86.18, 95.69)             # recency data
nytimes_rec_prob <- c(0.1597, 0.0778, 0.0557, 0.0445, 0.0419, 0.0361, 0.0338, 0.0295, 0.0272, 0.0237, 0.0209, 0.0165, 0.0151, 0.0124, 0.0118, 0.0107,
0.0098, 0.0092, 0.0083, 0.0071, 0.0062, 0.0052, 0.0052) # probability data
nytimes_recency <- data.frame(recency = nytimes_rec, prob = nytimes_rec_prob)           # create data frame
nytimes_mle_rec <- mle2(round(prob * 100, 0) ~ dbinom(prob = w1 * recency ^ w2, size = 100), data = nytimes_recency, start = list(w1 = 0.17, w2 = -0.7))
# calculate maximum likelihood estimator for power function
nytimes_pred_rec <- nytimes_mle_rec@coef[1] * (nytimes_rec) ^ nytimes_mle_rec@coef[2]   # create predicted values from MLE

# Generate NY Times headlines recency plot
nytimes_recency_plot <- xyplot(prob ~ recency, data = nytimes_recency, aspect = 1,      # plot data
  xlab = expression(paste("Words since last occurrence (", italic("t"), ")")),
  ylab = expression(paste("Probability of word occurrence (", italic("P"), ")")), ylim = c(-0.005, 0.185),
  par.settings = list(axis.text = list(cex = 2.25), par.xlab.text = list(cex = 2.75), par.ylab.text = list(cex = 2.75)),
  panel = function(x, y, ...) {
    panel.xyplot(x, y, type = "b", pch = 20, col = "black", cex = 2, lwd = 3)
    panel.text(x = 1, y = 0.1735, labels = "(a)", cex = 3.5)                                             # include subfigure label
    panel.lines(spline(nytimes_pred_rec  ~ nytimes_rec), col = "black")                                  # include fitted curve
    panel.text(70, 0.1585, label = expression(paste(italic("P"), " = 0.15", italic(t)^-0.73)), cex = 2.75)    # include text of fitted curve formula
  }
)

###################
## Human social contact data (Pachur et al. 2014)
###################
# Input and process data
human_social_rec_odds <- c(0.6749841548, 0.2667562085, 0.230387911, 0.1790514804, 0.1290453781, 0.1349800335, 0.2298386901, 0.0961911808, 0.0599465928,
0.0638485286, 0.0598481613, 0.0586801386, 0.0698224934, 0.0987088524, 0.0499042151, 0.0340669823, 0.0556853093, 0.0398437198, 0.0348255558, 0.041663257,
0.0584410567, 0.0385903784, 0.0321077701, 0.0290980865, 0.0249306312, 0.0241368483, 0.0344350147, 0.0563029054, 0.0300659544, 0.0265415731) # odds data
days1to30 <- 1:30           # create vector of recencies
human_social_rec_prob <- human_social_rec_odds / (1 + human_social_rec_odds)             # convert odds to probabilities
human_social_rec <- data.frame(pcontact = human_social_rec_prob, recency = days1to30)     # create data frame of probabilities of contact and recencies
human_social_rec_odds_fit <- 0.63 * days1to30 ^ {-0.91}                                  # create existing power function for contact data
human_social_rec_prob_fit <- human_social_rec_odds_fit / (1 + human_social_rec_odds_fit)  # convert odds to probabilities
mle2(round(pcontact, 2) * 100 ~ dbinom(prob = w1 * recency ^ -w2, size = 100), data = human_social_rec, start = list(w1 = 0.63, w2 = 0.91)) # calculate
MLE on human social contact recency data

# Generate human social contact recency plot
human_social_rec_plot <- xyplot(pcontact ~ recency, data = human_social_rec, aspect = 1,  # plot data
  xlab = expression(paste("Days since last contact (", italic("t"), ")")),
  ylab = expression(paste("Probability of contact (", italic("P"), ")")), ylim = c(-0.02, 0.48),
  par.settings = list(axis.text = list(cex = 2.25), par.xlab.text = list(cex = 2.75), par.ylab.text = list(cex = 2.75)),
  panel = function(x, y, ...) {
    panel.xyplot(x, y, type = c("b"), pch = 20, col = "black", cex = 2, lwd = 3)
    panel.text(x = 1, y = 0.445, labels = "(b)", cex = 3.5)                                                      # include subfigure label
    panel.lines(spline(human_social_rec_prob_fit  ~ days1to30), col = "black")                                   # include fitted curve
    panel.text(x = 22.5, y = 0.406, label = expression(paste(italic("P"), " = 0.41", italic(t)^-0.75)), cex = 2.75)   # include text of fitted curve
```

```
formula
  }
)

###################
## Chimpanzee recency data
###################
# Calculate probability of contact at recencies of 0-12 days
rec <- 1:window_size    # create vector of recencies

observed_rec_all <- findRecency(observed_matrix, nas_allowed)                         # run recency analysis from C++ script
observed_rec_contacts <- observed_rec_all$rec_contacts[1:window_size]                 # extract number contacts at different recencies
observed_rec_observations <- observed_rec_all$rec_observations[1:window_size]         # extract total number of observations at different recencies
num_rec_windows <- sum(observed_rec_observations)                                     # calculate total number of windows used in recency analysis
observed_rec <- data.frame(binconf(observed_rec_contacts, observed_rec_observations)) # calculate probability of contact and confidence intervals for
binomial probabilities
observed_rec$rec <- rec                                                               # include recencies in data frame
names(observed_rec) <- c("pcontact", "lower", "upper", "rec")                         # rename columns

# Calculate parameter estimates and log-likelihood for power function
mle_pwr_rec <- mle2(round(pcontact * 100, 0) ~ dbinom(prob = w1 * rec ^ w2, size = 100), data = observed_rec, start = list(w1 = 0.7, w2 = -0.7))  #
calculate maximum likelihood estimator for power function
pred_pwr_rec <- mle_pwr_rec@coef[1] * (rec) ^ mle_pwr_rec@coef[2]   # create predicted values from MLE
rec_coef1 <- round(mle_pwr_rec@coef[1], 2)                          # extract coefficient
rec_coef2 <- round(mle_pwr_rec@coef[2], 2)                          # extract coefficient
# Calculate parameter estimates and log-likelihood for exponential function
mle_exp_rec <- mle2(round(pcontact * 100, 0) ~ dbinom(prob = w1 * exp(w2 * rec), size = 100), data = observed_rec, start = list(w1 = 0.85, w2 = -0.25)) #
calculate maximum likelihood estimator for exponential function
pred_exp_rec <- mle_exp_rec@coef[1] * exp(mle_exp_rec@coef[2] * rec)  # create predicted values from MLE
# Calculate AICc
pwr_AICc_rec <- AICc(mle_pwr_rec, nobs = 100, k = 2)    # calculate corrected AICc
exp_AICc_rec <- AICc(mle_exp_rec, nobs = 100, k = 2)    # calculate corrected AICc
# Compare models
AICcs_rec <- c(pwr_AICc_rec, exp_AICc_rec)  # create vector of AICcs
minAICc_rec <- min(AICcs_rec)               # find minimum AICc
deltas_rec <- AICcs_rec - minAICc_rec       # calculate AIC differences
ws_rec <- (exp(-0.5 * deltas_rec)) / (sum(exp(-0.5 * deltas_rec)))  # calculate likelihoods of models given the data
evid_ratio_rec <- ws_rec[1] / ws_rec[2]     # calculate evidence ratio

# Generate chimpanzee recency plot
chimp_recency_plot <- xYplot(Cbind(pcontact, lower, upper) ~ rec, data = observed_rec, # plot data
  type = "b", cex = 1.75, pch = 20, col = "black", aspect = 1,
  xlim = c(-0.5, window_size + 1), ylim = c(-0.05, 0.75),
  xlab = expression(paste("Days since last contact (", italic("t"), ")")),
  ylab = expression(paste("Probability of contact (", italic("P"), ")")),
  par.settings = list(axis.text = list(cex = 2.25), par.xlab.text = list(cex = 2.75), par.ylab.text = list(cex = 2.75)),
  offset = unit(0.25,'inches'),
  panel = function(...) {
   panel.xYplot(lwd = 3, ...)
   panel.text(x = 0.5, y = 0.695, labels = "(c)", cex = 3.5)  # include subfigure label
   panel.lines(spline(pred_pwr_rec ~ rec), col = "black")     # include fitted curve
   panel.abline(h = mean_contact_prob, lty = 2)               # plot regression line
   panel.text(11.3, 0.63, label = substitute(paste(italic("P"), " = 0.59", italic(t)^-0.94)), cex = 2.75) # include fitted curve formula
   # panel.text(9, 0.785, label = substitute(paste(italic("P"), " = ", w1, italic("t "), " "), env = list(w1 = rec_coef1)), cex = 2.75)
   # panel.text(12, 0.8, label = (rec_coef2), cex = 2)
  }
)

## Print all recency plots
png(filename = "recency.png", width = 2000, height = 700)         # create PNG file
print(nytimes_recency_plot, split = c(1, 1, 3, 1), more = TRUE)   # plot human NY Times headlines data
```

```
print(human_social_rec_plot, split = c(2, 1, 3, 1), more = TRUE)  # plot human social contact data
print(chimp_recency_plot, split = c(3, 1, 3, 1), more = FALSE)    # plot chimpanzee social contact data
dev.off()                                                          # close PNG file

###################################################
### Memory analysis
###################################################
###################
## Human memory analysis
###################
# Input and process data
human_memory <- data.frame(recency = c(0.33, 1, 8.8, 24, 48, 144, 744), savings = c(58.2, 44.2, 35.8, 33.7, 27.8, 25.4, 21.1))  # human memory data from
Ebbinghaus (1913)
# Calculate parameter estimates and log-likelihood for power function
memory_times <- 0.15:750                            # create vector of retention times
memory_retention <- 47.56 * memory_times ^ - 0.126  # create vector of retention percentages based on Anderson & Schooler (1991) parameter estimates

human_pwr_mem <- mle2(round(savings, 0) ~ dbinom(prob = w1 * recency ^ w2, size = 100), data = human_memory, start = list(w1 = 0.05, w2 = -0.013))    #
calculate maximum likelihood estimator for power function

# Generate human memory plot
human_memory_plot <- xyplot(savings ~ recency, data = human_memory, aspect = 1, # plot data
  xlab = expression(paste("Hours of delay (", italic("t"), ")")),
  ylab = expression(paste("Percent savings (", italic("P"), ")")),
  ylim = c(18:65),
  par.settings = list(axis.text = list(cex = 2), par.xlab.text = list(cex = 3), par.ylab.text = list(cex = 3)),
  panel = function(x, y, ...) {
    panel.xyplot(x, y, type = "p", pch = 20, lwd = 2, col = "black", cex = 3)
    panel.text(x = -4, y = 62.5, labels = "(a)", cex = 2.95)                                              # include subfigure label
    panel.lines(spline(memory_retention ~ memory_times), col = "black")                                   # include fitted curve
    panel.text(550, 60, label = substitute(paste(italic("P"), " = 0.48 ", italic(t)^-0.13)), cex = 2.75)  # include fitted curve formula
  }
)

###################
## Chimpanzee memory analysis
###################
# Input and process data
mem_time <- c(2, 10, 20, 40, 2, 10, 20, 40, 4:10, 4:10, 4:9, rep(seq(5, 20, 5), each = 4), c(5, 10, 15))            # nonhuman retention times (Nissen et
al. 1938, Finch 1942)
mem_retention <- c(92, 81, 66, 60, 89, 80, 72, 62, 80.6, 67, 74, 80, 69.2, 61, 66.1, 80.7, 74.5, 76.4, 67.9, 71.4, 69.6, 63.1, 78.2, 81.9, 81.2, 75.4,
78, 81.7, 74.7, 67.2, 84.5, 70.1, 65.4, 62.5, 77.9, 70.2, 62.4, 69.8, 74.9, 79.6, 65.2, 69.9, 84.7, 80.2, 64.8, 64.8, 67)   # nonhuman retention
percentages (Nissen et al. 1938, Finch 1942)
chimp_memory <- data.frame(time = mem_time, retention = mem_retention)  # create data frame
chimp_memory_mean <- aggregate(retention ~ time, chimp_memory, mean)    # calculate means for each retention time
times <- 1.25:40                                                        # create vector of retention times

# Calculate parameter estimates and log-likelihood for power function
chimp_pwr_mem <- mle2(round(retention, 0) ~ dbinom(prob = w1 * time ^ w2, size = 100), data = chimp_memory_mean, start = list(w1 = 0.7, w2 = -0.7)) #
calculate maximum likelihood estimator for power function
chimp_pred_pwr_mem <- chimp_pwr_mem@coef[1] * (times) ^ chimp_pwr_mem@coef[2] # create predicted values from MLE
# Calculate parameter estimates and log-likelihood for exponential function
chimp_exp_mem <- mle2(round(retention, 0) ~ dbinom(prob = w1 * exp(w2 * time), size = 100), data = chimp_memory_mean, start = list(w1 = 0.85, w2 =
-0.25)) # calculate maximum likelihood estimator for exponential function
chimp_pred_exp_mem <- chimp_exp_mem@coef[1] * exp(chimp_exp_mem@coef[2] * times)  # create predicted values from MLE
# Calculate AICc
chimp_pwr_AICc_mem <- AICc(chimp_pwr_mem, nobs = 100, k = 2)    # calculate corrected AICc
chimp_exp_AICc_mem <- AICc(chimp_exp_mem, nobs = 100, k = 2)    # calculate corrected AICc
# Compare models
chimp_AICcs_mem <- c(chimp_pwr_AICc_mem, chimp_exp_AICc_mem)    # create vector of AICcs
chimp_minAICc_mem <- min(chimp_AICcs_mem)                       # find minimum AICc
```

```
chimp_deltas_mem <- chimp_AICcs_mem - chimp_minAICc_mem         # calculate AIC differences
chimp_ws_mem <- (exp(-0.5 * chimp_deltas_mem)) / (sum(exp(-0.5 * chimp_deltas_mem)))  # calculate likelihoods of models given the data
chimp_evid_ratio_mem <- chimp_ws_mem[1] / chimp_ws_mem[2]       # calculate evidence ratio

# Generate chimpanzee memory plot
chimp_memory_plot <- xyplot(retention ~ time, data = chimp_memory_mean, aspect = 1, # plot data
  xlab = expression(paste("Seconds of delay (", italic("t"), ")")),
  ylab = expression(paste("Percent correct (", italic("P"), ")")),
  xlim = c(-1, 42), ylim = c(58, 97),
  par.settings = list(axis.text = list(cex = 2), par.xlab.text = list(cex = 3), par.ylab.text = list(cex = 3)),
  panel = function(x, y, ...) {
    panel.xyplot(x, y, type = "p", pch = 20, lwd = c(1.5, 2.5, rep(1.5, 3)), col = "black", cex = 3, ...)
    panel.text(x = 1.5, y = 95, labels = "(b)", cex = 2.95)                                           # include subfigure label
    panel.lines(spline(chimp_pred_pwr_mem * 100 ~ times), col = "black")                             # include fitted curve
    panel.text(29, 92.85, label = substitute(paste(italic("P"), " = 0.95 ", italic(t)^-0.12)), cex = 2.75)  # include fitted curve formula
  }
)

## Print memory plots
png(filename = "memory.png", width = 1200, height = 600)          # create PNG file
print(human_memory_plot, split = c(1, 1, 2, 1), more = TRUE)      # plot human memory data
print(chimp_memory_plot, split = c(2, 1, 2, 1), more = FALSE)     # plot nonhuman memory data
dev.off()                                                         # close PNG file
```

```
//////////////////////////////
// stevens_etal_frequency.cpp
//////////////////////////////

#include <Rcpp.h>


using namespace Rcpp ;
// [[Rcpp::export]]
SEXP findFrequency(NumericMatrix cooccur_matrix, int windowsize, int nas_allowed){
  int days = cooccur_matrix.ncol();
  int num_pairs = cooccur_matrix.nrow();
  NumericVector current_window(windowsize + 1);           // array that includes actual window from cooccur matrix
  int sum_contact;                                        // total number of contacts within a window
  NumericVector contacts(days);                           // number of observations of each recency in which there is a contact
  NumericVector observations(days);                       // number of observations of each recency


  for(int i = 0; i < num_pairs; i++) {                    // for each pair
    for(int j = 0; j < (days - windowsize); j++) {        // for each day available for window size
      for(int k = 0; k < (windowsize + 1); k++) {         // for each element in the window
        current_window[k] = cooccur_matrix(i, j + k);     // assign current window
        if(k < windowsize) {                              // if this is in the window
          if(!R_IsNA(current_window[k])) {                // if the element is not NA
            sum_contact = sum_contact + current_window[k];// calculate total number of contacts (frequency) in that window
          }
        } else {                                          // if this is the contact day after the window
          // count the number of NAs in the window
          int na_counter = 0;                             // initiate the NA counter
          for(int i = 0; i < current_window.size(); i++) { // for each element in the window
            if(R_IsNA(current_window[i])) {               // if the current element is NA
              na_counter++;                               // increment the NA counter
            }
          }
          int num_nas = na_counter;                       // assign the number of NAs to num_nas
          if(num_nas < nas_allowed + 1) {                 // if there have been less than nas_allowed NAs in the window
            observations[sum_contact]++;                  // increment observation counter for that frequency
            if(current_window[k] == 1) {                  // if the contact day element is 1
              contacts[sum_contact]++;                    // increment the number of contacts for that frequency
            }
          }
        }
      }
      sum_contact = 0;                                    // reset number of contacts in the window
    }
  }
  return Rcpp::List::create(Rcpp::Named("freq_contacts") = contacts, Rcpp::Named("freq_observations") = observations);
}
```

```cpp
//////////////////////////////
// stevens_etal_frequency.cpp
//////////////////////////////

#include <Rcpp.h>

using namespace Rcpp ;
// [[Rcpp::export]]
SEXP findRecency(NumericMatrix cooccur_matrix, int na_allowed){
  int days = cooccur_matrix.ncol();
  int num_pairs = cooccur_matrix.nrow();
  int contact_check;                          // flag to check whether contact has occurred for that row
  int recency_counter;                        // counts intervening days to calculate recency
  NumericVector contacts(days);               // number of observations of each recency in which there is a contact
  NumericVector observations(days);           // number of observations of each recency

  for(int i = 0; i < num_pairs; i++) {        // for each pair
    int contact_check = 0;                    //  reset contact check for new pair
    int recency_counter = 0;                  //  reset recency counter
    int na_counter = 0;                       // reset NA counter
    for(int j = 0; j < days; j++) {           //  for each day
      if(!R_IsNA(cooccur_matrix(i, j)) | na_counter <= na_allowed) {          //   if the element is not NA or if the NA counter is less than the number
of NAs allowed
        if(contact_check == 0) {              //    if contact has not occurred before
          if(cooccur_matrix(i, j) == 1) {     //     if current observation is a contact
            contact_check = 1;                //      assign contact check flag to signal that this pair has had previous contact
          }
        } else {                              //    if contact has occurred before
          observations[recency_counter]++;    //     incremement number of observations for appropriate recency
          if(cooccur_matrix(i, j) == 1) {     //     if current observation is a contact
            contacts[recency_counter]++;      //      increment number of instances for appropriate recency
            recency_counter = 0;              //      reset recency counter
          } else {                            //     if the current observation is not a contact
            recency_counter++;                //      increment recency counter
          }
        }
      } else {                                //   if the element is NA
        na_counter++;                         //    increment NA counter
        recency_counter = 0;                  //    reset recency counter
        contact_check = 0;                    //    reset recency counter
      }
    }
  }
  return Rcpp::List::create(Rcpp::Named("rec_contacts") = contacts, Rcpp::Named("rec_observations") = observations);
}
```
